# Morphology-Dependent Antibacterial Activity of Cu_2-x_S Nanostructures: Nanoplates Versus Superparticles

**DOI:** 10.3390/nano16100636

**Published:** 2026-05-20

**Authors:** Hui Zhu, Mengzhe Zhao, Yang Chao, Jun Yao, Qin Yu, Na Sun

**Affiliations:** 1 School of Modern Fashion, Anhui University of Applied Technology, Hefei 230011, China; yaojun@uta.edu.cn (J.Y.); yuqin@uta.edu.cn (Q.Y.); 2 Key Laboratory of Functional Molecular Solids, Ministry of Education College of Chemistry and Materials Science, Anhui Normal University, Wuhu 241000, China; zhao20231@outlook.com (M.Z.); chaoyang982310@163.com (Y.C.); 3 Huzhou Key Laboratory of Green Energy Materials and Battery Cascade Utilization, School of Intelligent Manufacturing, Huzhou College, Huzhou 313000, China

**Keywords:** Cu_2-x_S, morphology-dependent, antibacterial performance, photothermal conversion efficiency, ROS, Cu^2+^ release

## Abstract

Non-stoichiometric copper sulfide (Cu_2-x_S) nanomaterials are promising antibacterial agents, but the role of morphology in regulating their bactericidal performance remains poorly understood. Herein, we rationally design two types of Cu_2-x_S nanostructures, namely nanoplates (NPs) and superparticles (SPs). Both materials were prepared via a ligand-directed synthesis method with the comparable sizes, surface ligands, and crystal phase. The antibacterial behaviors of Cu_2-x_S NPs and Cu_2-x_S SPs against *Escherichia coli* (*E. coli*) and *Staphylococcus aureus* (*S. aureus*) were investigated under dark and 808 nm near-infrared (NIR) light irradiation. The results showed that under NIR light irradiation, Cu_2-x_S SPs exhibit a markedly higher bactericidal efficiency against both *E. coli* and *S. aureus* than Cu_2-x_S NPs, leading to almost complete eradication of bacterial colonies. Notably, *S. aureus* shows more sensitive than *E. coli*, and significant growth inhibition is observed even in the absence of laser irradiation. Mechanistic investigations reveal that hierarchical assembly of primary nanoparticles in SPs can promote multiple internal light scatterings, thereby significantly enhancing light harvesting efficiency and further improving the photothermal conversion efficiency. In addition, the SPs exhibited higher peroxidase-like activity, resulting in enhanced reactive oxygen species (ROS) generation and aggravated oxidative damage, and the accelerated Cu^2+^ release kinetics strengthens ionic toxicity.

## 1. Introduction

The rapid and persistent spread of multidrug-resistant bacterial infections poses severe challenges to conventional antibiotic therapies [1,2]. It is urgent to develop alternative antibacterial agents. In recent years, nanomaterials have emerged as promising alternatives to traditional small-molecule antibiotics [3]. Nanomaterials exhibit therapeutic potential that transcends that of traditional agents, owing to their precisely tailorable physicochemical properties as follows: size, surface functionality, and morphological architecture [4,5,6].

Among the various nanomaterial candidates, non-stoichiometric copper sulfides (Cu_2-x_S) have attracted widespread attention [7,8,9,10,11]. The partial copper deficiency in Cu_2-x_S endows it with strong near-infrared (NIR) localized surface plasmon resonance (LSPR) property, enabling efficient photothermal therapy (PTT). Meanwhile, the Cu^+^/Cu^2+^ redox couple in these materials can trigger a Fenton-like reaction, which can produce reactive oxygen species (ROS) [12,13,14,15]. These combined properties make Cu_2-x_S a promising candidate for developing multifunctional antibacterial platforms, particularly for light-triggered sterilization. However, despite the rapid progress in Cu_2-x_S-based antibacterial systems, most existing studies have mainly focused on compositional optimization, defect engineering, or surface functionalization, whereas the influence of morphology itself remains insufficiently clarified. The antibacterial performance of Cu_2-x_S is determined by a complex interplay of factors [16,17,18,19,20]. Although parameters such as size, surface chemistry, chemical doping and surface functionalization have been broadly investigated [21,22,23], elucidating the specific contribution of geometric morphology remains highly challenging. This difficulty primarily arises from the fact that altering the shape of a nanomaterial often inevitably changes its size, crystal phase, surface ligands, and so on [24,25,26]. Thus, it is difficult to isolate morphology as the sole variable.

To better position the present study, representative CuS-based antibacterial systems reported in the literature were compared, with particular emphasis on morphology-dependent effects (Table S1). Previous studies have shown that reducing particle size, introducing porous/amorphous structures, and tailoring crystal shape can all improve the antibacterial activity of CuS materials by enhancing photothermal conversion, ROS generation, and/or Cu^2+^ release. For example, ultrasmall CuS nanodots exhibited stronger bactericidal performance than larger nanoparticles [8], while CuS nanosheets and nanoparticles outperformed microspheres in light-driven antibacterial assays [18]. Porous amorphous CuS-based nanoparticles have also been reported to combine improved light absorption with enhanced ROS-related killing [21]. However, in most of these studies, multiple physicochemical parameters changed simultaneously, making it difficult to distinguish the intrinsic contribution of morphology from those of size, surface area, colloidal stability, or surface chemistry. In comparison, the present work provides a more controlled evaluation of the morphology effect because Cu_2-x_S nanoplates and superparticles were designed with comparable size, surface ligand environment, and crystal phase, thereby minimizing interference from other physicochemical factors. Under these matched conditions, Cu_2-x_S superparticles still exhibited substantially enhanced antibacterial activity relative to nanoplates, indicating that morphology plays an important role in regulating the antibacterial performance of Cu-based nanomaterials. In this sense, our study complements previous reports and provides a clearer perspective for understanding morphology-dependent structure–activity relationships than systems relying primarily on compositional modification or surface functionalization.

**Scheme 1:**
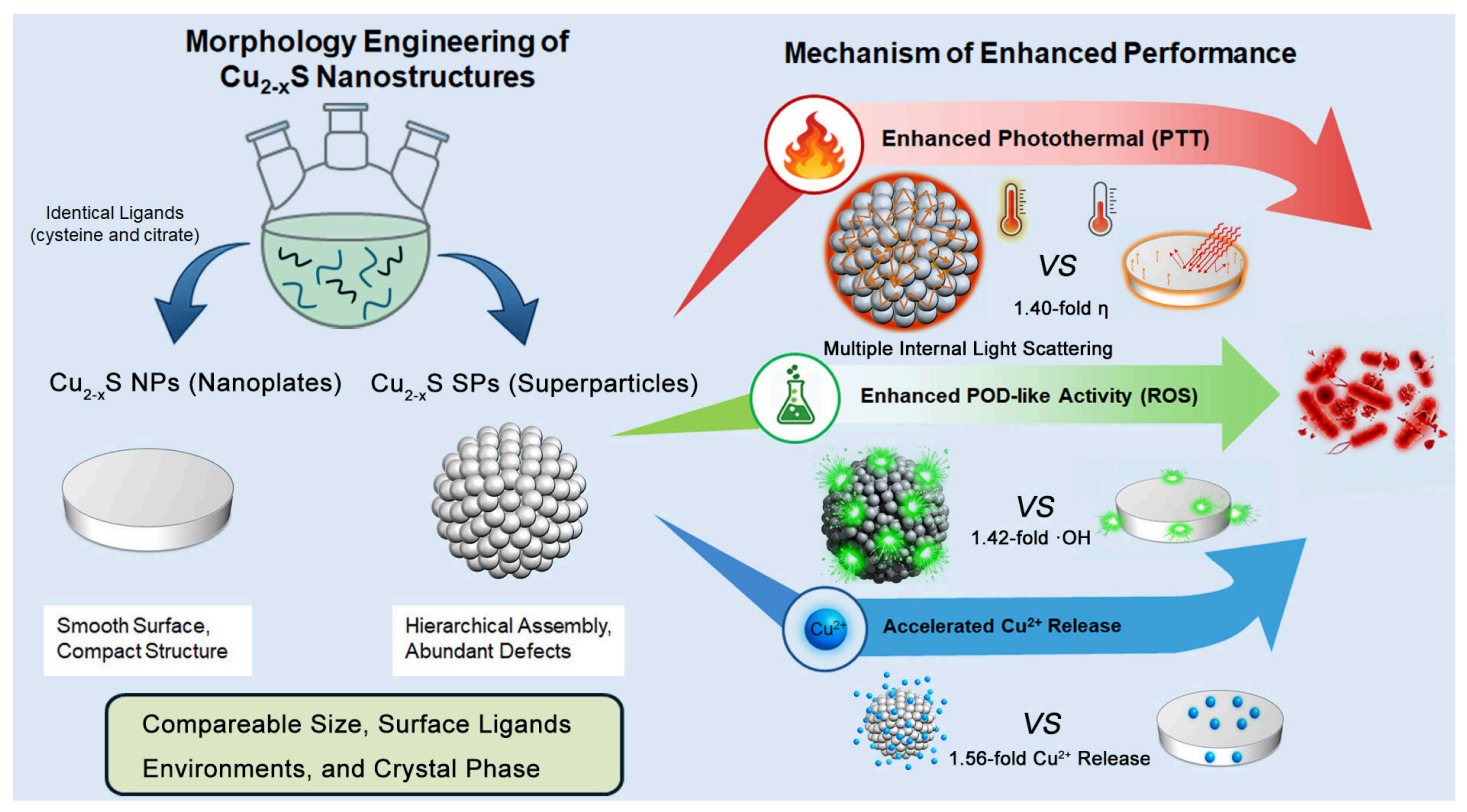
Schematic illustration of the ligand-directed formation of Cu_2-x_S NPs and hierarchically assembled SPs, as well as the proposed mechanism underlying the enhanced antibacterial activity of Cu_2-x_S SPs.

Based on the previous work [27], we employ a ligand-directed synthetic strategy to fabricate two morphologies of Cu_2-x_S nanostructures, namely nanoplates (NPs) and hierarchical superparticles (SPs). In this strategy, the same ligand environment (cysteine and sodium citrate) was used to regulate the nucleation, growth, and assembly behavior of Cu_2-x_S during synthesis, thereby minimizing variations in size, surface chemistry, and crystal phase between the two samples. The Cu_2-x_S SPs are formed through the self-assembly of primary nanocrystals under these ligand-directed conditions. Their detailed formation process and structural evolution have been systematically reported in our previous study [27]; therefore, the present work focuses on morphology-dependent antibacterial behavior rather than the assembly mechanism itself. These two nanostructures were systematically characterized via transmission electron microscopy (TEM), X-ray diffraction (XRD), X-ray photoelectron spectroscopy (XPS), Fourier transform infrared spectroscopy (FTIR), and ζ-potential measurements. The characterization results confirmed that Cu_2-x_S NPs and Cu_2-x_S SPs exhibited comparable sizes, surface ligands environments (cysteine and sodium citrate), and crystal phase, but different morphologies. Plate counting antibacterial assay revealed an obvious morphology-dependent antibacterial activity of the two nanostructures. Furthermore, three core factors, including photothermal conversion efficiency, peroxidase (POD)-like ROS burst, and cumulative Cu^2+^ release kinetics, were compared to clarify the factors contributing to the differing antibacterial activities of the two nanostructures against Gram-negative (*Escherichia coli*, *E. coli*) and Gram-positive (*Staphylococcus aureus*, *S. aureus*) bacteria (Scheme 1). This study systematically demonstrated that the hierarchical structure significantly enhanced the antibacterial effect, providing a useful strategy for designing the structure of a new generation of highly efficient antibacterial nanomaterials.

## 2. Materials and Methods

### 2.1. Chemicals and Materials

CuSO_4_·5H_2_O, NaOH, sodium citrate and hydrogen peroxide (H_2_O_2_, 30%) were purchased from Shanghai Reagent Company (Shanghai, China). Cysteine and thioacetamide (TAA) were acquired from Aladdin. 3,3’,5,5’-tetramethylbenzidine (TMB) was purchased from Macklin. Dulbecco’s modified Eagle medium (DMEM), penicillin-streptomycin, and fetal bovine serum (FBS) were purchased from Gibco Invitrogen. Cell counting kit-8 (CCK-8) and bacterial live/dead staining kit (DMAO/PI) were purchased from Beyotime Biotechnology (Shanghai, China). Luria–Bertani (LB) broth medium and LB broth agar medium were obtained from Sangon Biotech Co., Ltd. (Shanghai, China). Milli-Q deionized water was used to prepare all solutions.

### 2.2. Characterizations

Transmission electron microscopy (TEM) images were taken by a HT-7800 electron microscope (Hitachi, Japan). High-resolution TEM (HRTEM) images were carried out on a Tecnai F20 TEM (FEI, Hillsboro, OR, USA). Extinction spectra were recorded by a U-2910 spectrometer (Hitachi, Tokyo, Japan). The hydrodynamic particle sizes and ζ-potential values were examined on a Zetasizer Nano ZS series instrument (Malvern Instruments Co., Ltd., Malvern, UK). Fourier transform infrared spectroscopy (FTIR) spectra (4000–1000 cm^−1^) were measured by a Magna-560 spectrometer (Nicolet, Madison, WI, USA). The amounts of Cu were recorded by inductively coupled plasma mass spectrometry (ICP-MS, Thermo Elemental, Winsford, UK). X-ray photoelectron spectroscopy (XPS) was characterized with the ESCALAB 250Xi photoelectron spectrometer (Thermo Fisher Scientific, Waltham, MA, USA). X-ray powder diffraction (XRD) patterns were obtained on a Shimadzu XRD-6000 X-ray diffractometer equipped (Shimadzu, Kyoto, Japan). Photothermal irradiation was conducted by an 808 nm laser with 0.75 W cm^−2^ (CNI, Changchun, China) and the temperature was measured by an infrared thermal imaging instrument (FLIR, A65, Wilsonville, OR, USA). Cell imaging was performed using an automated inverted optical microscope (Olympus IX83, Tokyo, Japan). Fluorescent images of cells were taken by a confocal laser scanning fluorescence microscope (TCS SP8, Leica, Wetzlar, Germany).

### 2.3. Fabrication of the Cu_2-x_S NPs

First, sodium citrate solution (0.1 M, 1 mL) and deionized water (40 mL) were introduced into a three-necked flask. The above solution was deaerated by bubbling nitrogen for 20 min under magnetic stirring to remove dissolved oxygen. Next, pre-mixed solution of cysteine (0.1 M, 3 mL) and CuSO_4_ (0.4 M, 125 μL) was added. Then, NaOH (0.5 M) solution was added into the above solution to adjust the pH to 10.0, followed by stirring for 10 min. Subsequently, TAA (1 mL, 0.01 M) was introduced into the reaction mixture. Finally, the resulting solution was heated to 70 °C and refluxed under a nitrogen atmosphere for 22 h. Monodisperse Cu_2-x_S NPs with an average size of 16.03 nm were obtained. The Cu_2-x_S NPs solution was purified using 8000–14,000 Da MWCO dialysis bags in double deionized water for 30 h.

### 2.4. Fabrication of the Cu_2-x_S SPs

First, citrate solution (0.1 M, 1 mL) and deionized water (40 mL) were introduced into a three-necked flask. The above solution was deaerated by bubbling nitrogen for 20 min under magnetic stirring to remove dissolved oxygen. Next, pre-mixed cysteine (0.1 M, 3 mL) and CuSO_4_ (0.4 M, 125 μL) was added into the above solution. Then, NaOH (0.5 M) solution was added into the above solution to adjust the pH to 10.0, follow by stirring for 10 min. Subsequently, TAA (1 mL, 0.01 M) was introduced into the reaction mixture. Thereafter, the resulting solution was heated to 70 °C and refluxed under a nitrogen atmosphere for 5 h. Next, the cysteine–CuSO_4_ mixture (cysteine, 3 mL, 0.1 M; CuSO_4_, 125 μL, 0.4 M) was injected, and the pH was readjusted to 10.0. Finally, the resulting reaction mixture was heated to 70 °C and refluxed under nitrogen atmosphere for 16 h. The Cu_2-x_S SPs solution was purified using 8000–14,000 Da MWCO dialysis bags in double deionized water for 24 h.

In both syntheses, the same ligand system consisting of cysteine and sodium citrate was employed to regulate the formation process of Cu_2-x_S nanostructures. This design was intended to minimize variations in surface ligand environment and crystal phase between the Cu_2-x_S NPs and Cu_2-x_S SPs, so that the effect of morphology on antibacterial performance could be more reliably evaluated.

### 2.5. Preparation of Cu_2-x_S NPs and Cu_2-x_S SPs in PBS

After dialysis purification, the Cu_2-x_S NPs and Cu_2-x_S SPs were freeze-dried to obtain solid powders. For experiments performed in PBS, the dried powders were redispersed in phosphate-buffered saline (PBS, pH 7.4) to the desired concentrations before use.

### 2.6. Cytotoxicity Assays

The viabilities of human umbilical vein endothelial cells (HUVECs) and mouse fibroblasts (3T3) cells treated with Cu_2-x_S NPs and Cu_2-x_S SPs were investigated using the CCK-8 assays. Cells were seeded into 96-well plates at a density of approximately 9 × 10^3^ cells per well and incubated for 12 h. The culture medium was then replaced with medium containing Cu_2-x_S NPs and Cu_2-x_S SPs at various concentrations (0, 7.5, 15, 30, 60, and 120 μg·mL^−1^). After 24 h of incubation, the medium was removed, and the cells were washed three times with PBS (pH 7.4). Next, 200 μL of culture medium supplemented with 5% CCK-8 was added to each well, and the cells were incubated at 37 °C for 3 h. Cell viability was finally measured on a microplate reader (Thermo Varioskan Flash, Waltham, MA, USA).

### 2.7. Antibacterial Assays

*E. coli* (Gram-negative) and *S. aureus* (Gram-positive) were cultured in LB broth at 37 °C to logarithmic phase (106 CFU mL^−1^). Bacteria were incubated with Cu_2-x_S NPs or SPs (120 μg mL^−1^) with/without 808 nm laser irradiation (0.75 W cm^−2^, 10 min). Colony-forming unit (CFU) counting and live/dead staining (DMAO/PI) were used to evaluate antibacterial efficiency.

### 2.8. POD-like Activity Assay

The generation of ROS (·OH) was measured using TMB-H_2_O_2_ colorimetric assay. Measurements were carried out in 2 mL of PBS (0.1 M, pH 7.4) containing Cu_2-x_S NPs and Cu_2-x_S SPs at the same concentration (120 μg mL^−1^), H_2_O_2_ (1 mM), and TMB (0.5 mM). The mixtures were incubated at 37 °C for 10 min, and the absorbance spectra were recorded using a UV–vis–NIR spectrometer (Hitachi, Tokyo, Japan).

### 2.9. The Release of Copper Ions

To monitor Cu^2+^ release kinetics, 200 μg of Cu_2-x_S NPs and Cu_2-x_S SPs were dispersed in 2 mL of PBS (pH 7.4), respectively. The above two dispersions were dialysed at 37 °C. The release medium was taken out at different time points (0, 2, 6, 12 and 24 h); an aliquot of the release medium was collected and replaced with an equal volume of fresh PBS. The concentration of Cu^2+^ in the collected medium was determined by ICP-MS.

## 3. Results

### 3.1. Structural and Morphological Characterization

**Figure 1:**
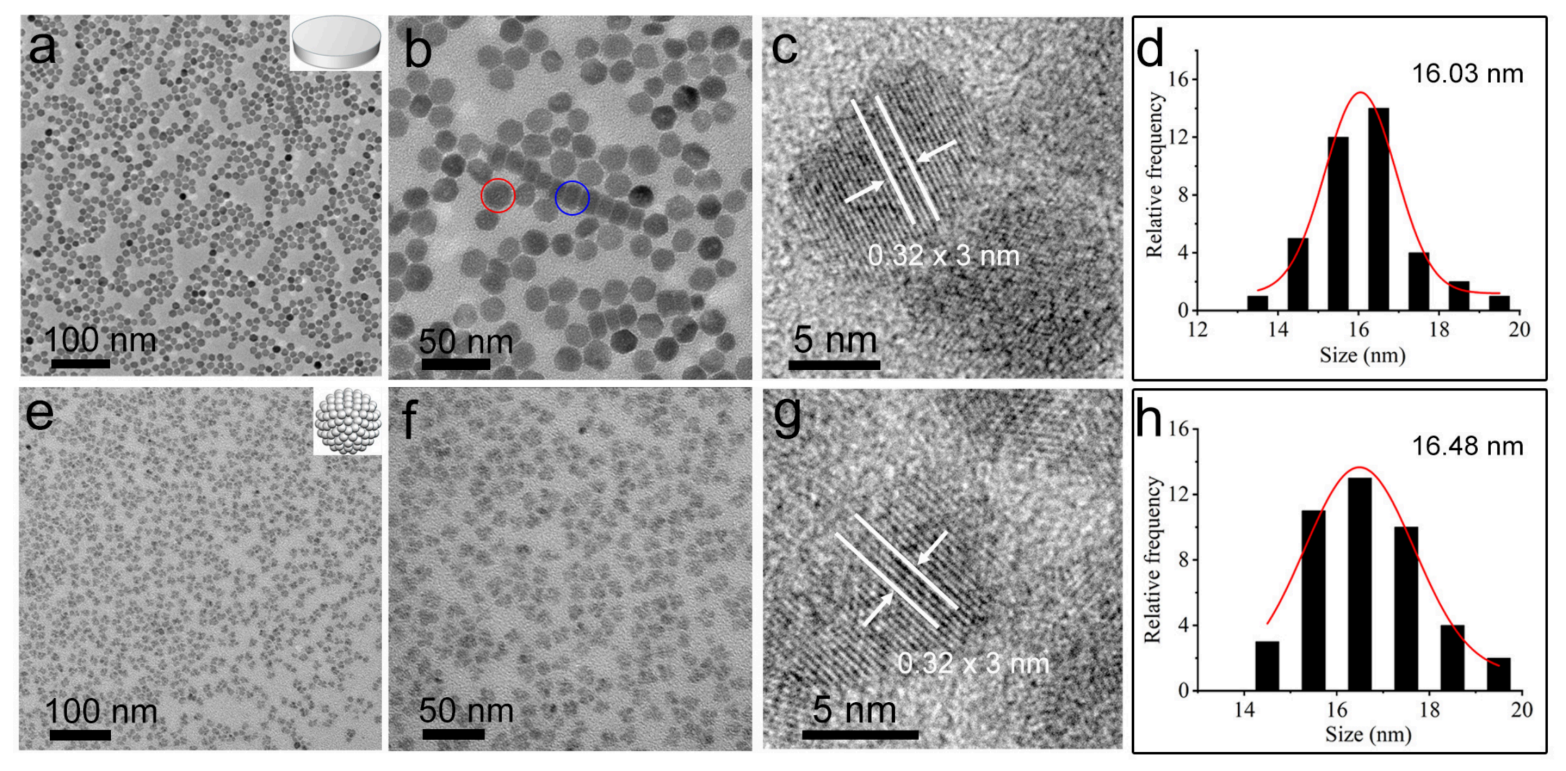
Morphological characteristics of Cu_2-x_S NPs and Cu_2-x_S SPs. (**a**,**b**) TEM images of Cu_2-x_S NPs at different magnifications, the red and blue circles indicate the circular and rectangular morphologies, respectively; (**c**) HRTEM image of Cu_2-x_S NPs; (**d**) size distribution of Cu_2-x_S NPs; (**e**,**f**) TEM images of Cu_2-x_S SPs at different magnifications; (**g**) HRTEM image of Cu_2-x_S SPs; (**h**) size distribution of Cu_2-x_S SPs.

Cu_2-x_S NPs and Cu_2-x_S SPs were synthesized via a ligand-directed approach based on our previous work [27]. The two materials were prepared in a one-pot aqueous system using the same ligand type, copper and sulfur precursors, and reaction temperature (70 °C), while varying only the sodium citrate concentration. Figure 1a,b show TEM images of the as-prepared Cu_2-x_S NPs at different magnifications. As illustrated in Figure 1a, the products exhibit a typical monodisperse plate-shaped morphology. Most particles lie flat on the substrate, while only a small number are oriented on their sides. There are two typical morphologies, namely circular and rectangular (marked by red and blue circles, respectively). The average diameter of the circular structure is 16.03 nm (Figure 1d), and the thickness of the rectangular structure is 6.75 nm (Figure S1). According to the dynamic light scattering (DLS) measurements, the average hydrodynamic diameter of Cu_2-x_S NPs is 20.11 nm (Figure S2a), which is slightly larger than the size measured by TEM. This discrepancy can be attributed to the hydration shell formed around the Cu_2-x_S NPs, as well as the inherent bias of DLS toward larger particles. Subsequently, by carefully tuning interparticle interactions through the sodium citrate concentration, the primary nanoparticles were induced to assemble into monodisperse hierarchical superparticles. Notably, although the overall dimensions of the Cu_2-x_S SPs are comparable to those of the Cu_2-x_S NPs, the Cu_2-x_S SPs represent hierarchically assembled architectures composed of primary nanocrystals rather than single platelet-like nanostructures. Thus, the term “superparticle” in this work refers to the internal assembled structure rather than necessarily a much larger particle size. This structural assignment is consistent with our previous study on Cu_2-x_S supraparticle formation [27]. As shown in Figure 1e,f, Cu_2-x_S SPs were formed through self-assembly of primary nanoparticles, presenting a monodisperse rough spherical-like structure. The statistical size distribution of Cu_2-x_S SPs is 16.48 nm (Figure 1h). The hydrodynamic diameter of Cu_2-x_S SPs is 20.74 nm (Figure S2b). In Figure 1c,g, HRTEM images clearly show that the lattice plane spacings of both are 0.32 nm, corresponding to the (0015) crystal plane of the rhombohedral Cu_9_S_5_ (JCPDS No. 47-1748) [28].

**Figure 2:**
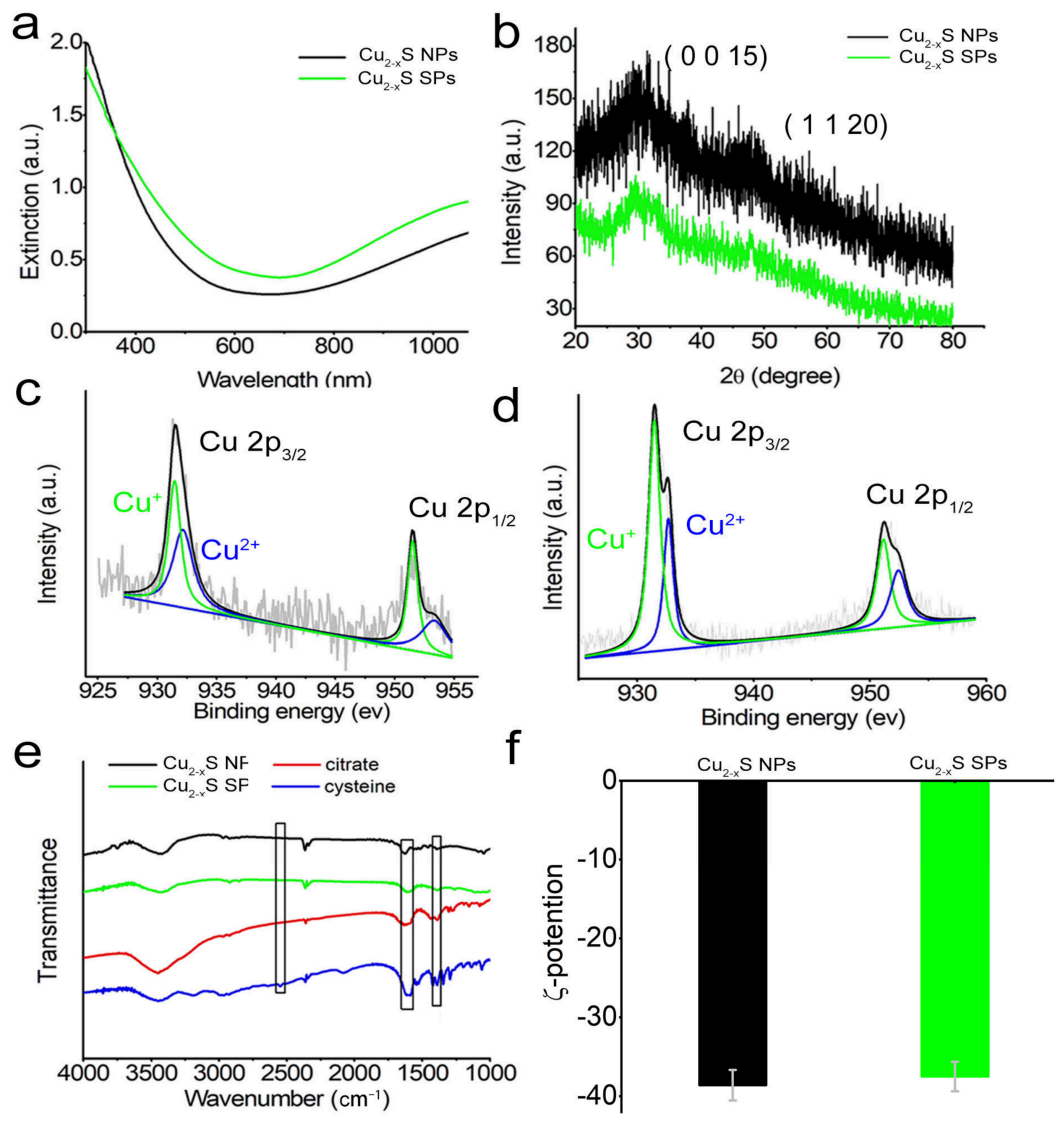
Structural and surface characterization of Cu_2-x_S NPs and Cu_2-x_S SPs. (**a**) Extinction spectra; (**b**) XRD patterns; (**c**,**d**) Cu 2p XPS spectra of NPs and SPs, respectively; (**e**) FTIR spectra; (**f**) ζ-potential distributions.

After confirming the morphology and size, the optical properties and structure features were further verified to ensure that morphology was the primary variable. As shown in Figure 2a, both Cu_2-x_S NPs and Cu_2-x_S SPs exhibit broad extinction characteristics in NIR regions, which corresponds to the LSPR originating from the collective oscillation of free holes driven by the electromagnetic field of incident light [29]. Moreover, at the same mass concentration, Cu_2-x_S SPs display a stronger absorption peak than Cu_2-x_S NPs. This enhancement effect is likely attributed to the hierarchical structure of Cu_2-x_S SPs, which promotes internal multiple light scattering, thereby significantly improving the overall light capture efficiency. Additionally, as described in Figure S3a,c, the extinction spectra of Cu_2-x_S NPs and Cu_2-x_S SPs were recorded at varying concentrations (7.5 to 120 μg mL^−1^). The extinction intensity at 808 nm increased linearly with the increase in their concentration (Figure S3b,d). This result confirms that both samples can maintain stable monodispersity in aqueous media and exhibit high anti-aggregation properties.

To investigate the crystal structure of Cu_2-x_S NPs and Cu_2-x_S SPs, XRD analyses were carried out. As shown in Figure 2b, the two samples exhibit distinctly broadened peaks, which is attributed to the small size of the products [30]. Despite the pronounced noise signals, the main diffraction peaks can be clearly indexed to the (0015) and (1120) planes and can be accurately indexed to the rhombohedral phase of Cu_9_S_5_ (JCPDS No. 47-1748), in agreement with the HRTEM characterization. To further investigate the composition and oxidation state of the surface elements, XPS was employed. The Cu 2p spectrum of Cu_2-x_S NPs (Figure 2c) can be fitted into the following four peaks: the signals at 931.4 eV and 951.7 eV correspond to Cu^2+^ (Cu 2p_3_/_2_ and Cu 2p_1_/_2_), while the peaks at 932.7 eV and 952.6 eV are assigned to Cu^+^ (Cu 2p_3_/_2_ and Cu 2p_1_/_2_). Similarly, the Cu 2p spectrum of Cu_2-x_S SPs exhibits four well-resolved peaks at 931.5 eV, 951.7 eV (Cu^2+^), and 932.6 eV, 952.5 eV (Cu^+^), respectively [28]. These results demonstrate that the surface copper species in both nanostructures exhibit very similar Cu^+^/Cu^2+^ oxidation state distributions.

As shown in Figure 2e, the absorption peak at 1630 cm^−1^ corresponds to the bending vibration of adsorbed water and surface hydroxyl groups (−OH), while the broad band at 3430 cm^−1^ is attributed to the O−H stretching vibrations of surface-adsorbed water and terminal −OH species [31]. Both nanostructures also display a characteristic band at 1350 cm^−1^ corresponding to C–N stretching. Crucially, the characteristic −SH stretching vibration of free cysteine (~2500 cm^−1^) is completely absent in the spectra of both Cu_2-x_S NPs and Cu_2-x_S SPs, confirming that cysteine is covalently anchored to the Cu_2-x_S surface via strong S–Cu bonds [32]. The above FTIR data unambiguously verify that both nanostructures were successfully modified with cysteine and sodium citrate, demonstrating their identical and effective surface functionalization. Finally, in Figure 2f, the purified Cu_2-x_S NPs and Cu_2-x_S SPs demonstrate highly comparable ζ-potential values of −38.6 mV and −37.5 mV, respectively. The ζ-potential values indicate that the two nanostructures have very similar surface charge densities and comparable ligand coverage. Taken together, the above results demonstrate that the Cu_2-x_S NPs and Cu_2-x_S SPs have comparable sizes, surface ligand environments, and the same crystal phase. Therefore, the differences observed in subsequent biological experiments can be mainly attributed to their distinct morphologies.

### 3.2. Biocompatibility

**Figure 3:**
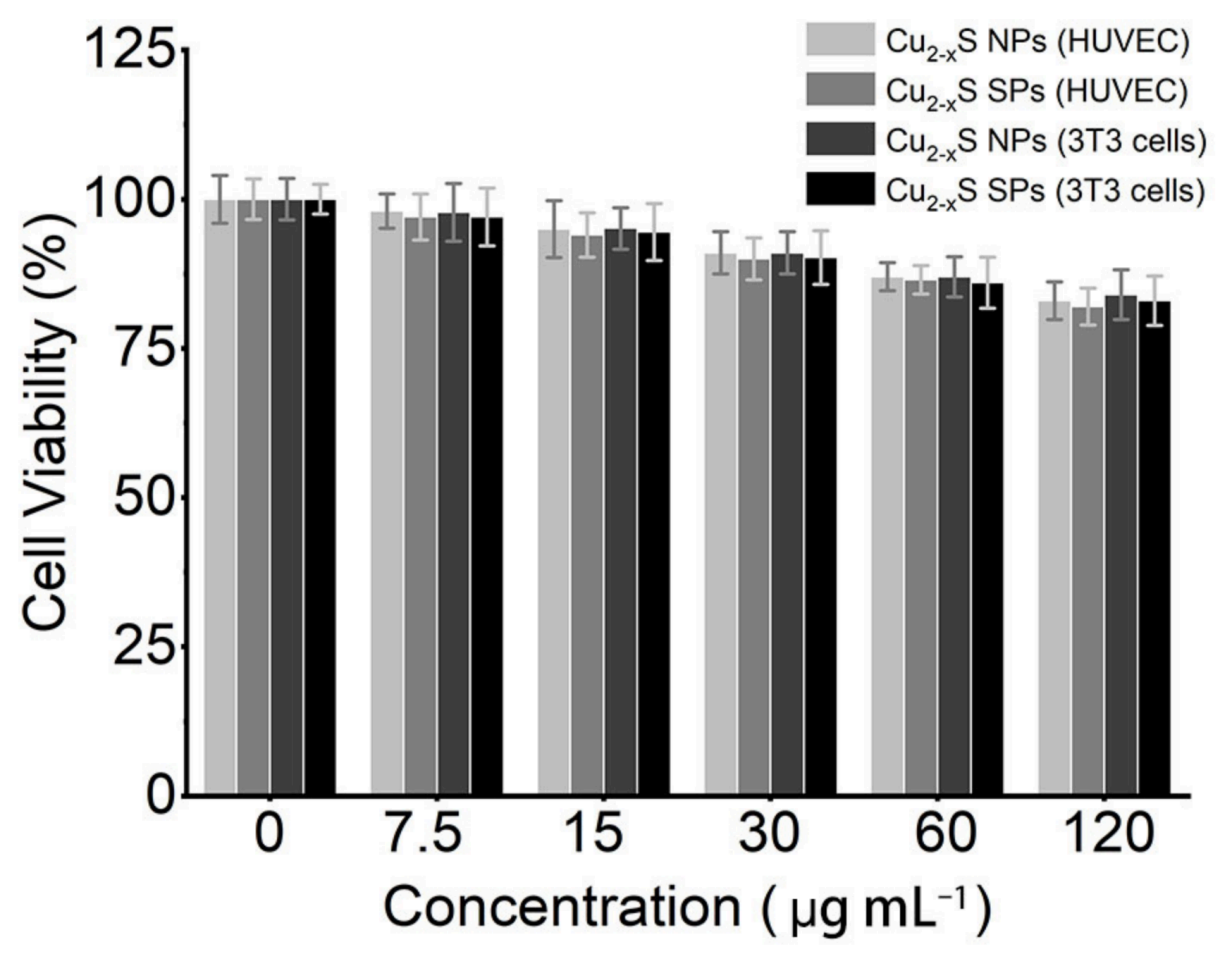
Cytotoxicity of HUVECs and 3T3 cells treated with Cu_2-x_S NPs and Cu_2-x_S SPs after 24 h incubation.

In practical antibacterial applications, ensuring the biological safety is essential. Therefore, the CCK-8 assays were employed to determine the viability of HUVECs and 3T3 cells treated with Cu_2-x_S NPs and Cu_2-x_S SPs. As shown in Figure 3, neither nanostructure exhibit significant cytotoxicity after 24 h incubation; even the concentration reached up to 120 μg mL^−1^, and the survival rates remained above 80%. These results indicate that both Cu_2-x_S NPs and Cu_2-x_S SPs exhibit acceptable in vitro cytocompatibility, providing preliminary support for their potential use in antibacterial-related biomedical applications.

### 3.3. Antibacterial Performance

To reveal the respective contributions of the nanomaterials and photothermal activation, we systematically evaluated their antibacterial efficacy using standard CFU assays. A comprehensive comparison was conducted against typical Gram-negative (*E. coli*) and Gram-positive (*S. aureus*) strains across the following six parallel groups: PBS, Cu_2-x_S NPs, and Cu_2-x_S SPs, each test with or without 808 nm NIR laser irradiation.

**Figure 4:**
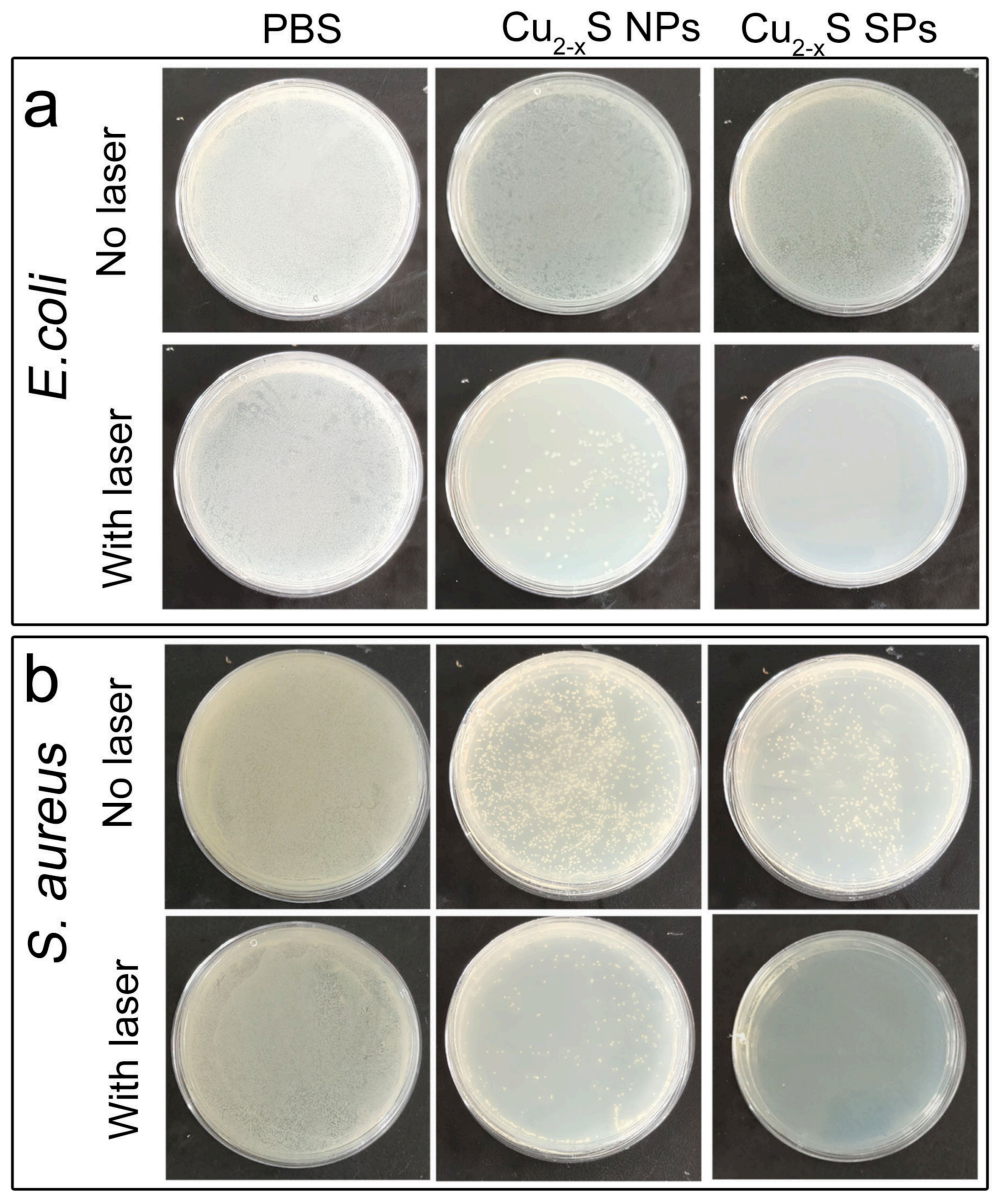
Antibacterial activity of Cu_2-x_S NPs and Cu_2-x_S SPs against (**a**) *E. coli* and (**b**) *S. aureus* under different treatments (PBS, PBS + laser, Cu_2-x_S NPs, Cu_2-x_S NPs + laser, Cu_2-x_S SPs, Cu_2-x_S SPs + laser).

The representative agar plate images are displayed in Figure 4. For *E. coli* (Figure 4a), dense bacterial colonies were observed in the PBS, Cu_2-x_S NPs, and Cu_2-x_S SPs groups under dark conditions. This phenomenon indicates that both nanostructures exhibited negligible intrinsic dark toxicity against *E. coli.* Upon 808 nm laser exposure, the PBS + laser group showed no obvious reduction in colony number, confirming that the laser treatment itself had little effect on bacterial viability. In contrast, the Cu_2-x_S NPs + laser group exhibited a significant decrease decreased in colony number, although substantial residual growth remained. In sharp contrast, the Cu_2-x_S SPs + laser group displayed almost completely eradicated the colonies, resulting in a clear agar plate. These results demonstrate that Cu_2-x_S SPs has an extremely significant photothermal-activated antibacterial effect against *E. coli.* compared with Cu_2-x_S NPs. *S. aureus* exhibited a different sensitivity characteristic from *E. coli* (Figure 4b). Even without laser irradiation (in the dark), Cu_2-x_S SPs exhibited a sparser distribution of bacteria compared to Cu_2-x_S NPs. This enhanced dark toxicity toward *S. aureus,* likely associated with the absence of an outer membrane barrier in Gram-positive bacteria, which may facilitate stronger interactions with released Cu^2+^, ROS, and the Cu_2-x_S SP surface [4]. Upon NIR irradiation, the number of bacterial colonies in Cu_2-x_S NPs group further decreased but still remained. In contrast, the Cu_2-x_S SPs + laser group achieved nearly complete elimination of *S. aureus*. These results indicate that Cu_2-x_S SPs possess superior antibacterial activity against both Gram-negative and Gram-positive bacteria, particularly against *S. aureus*.

**Figure 5:**
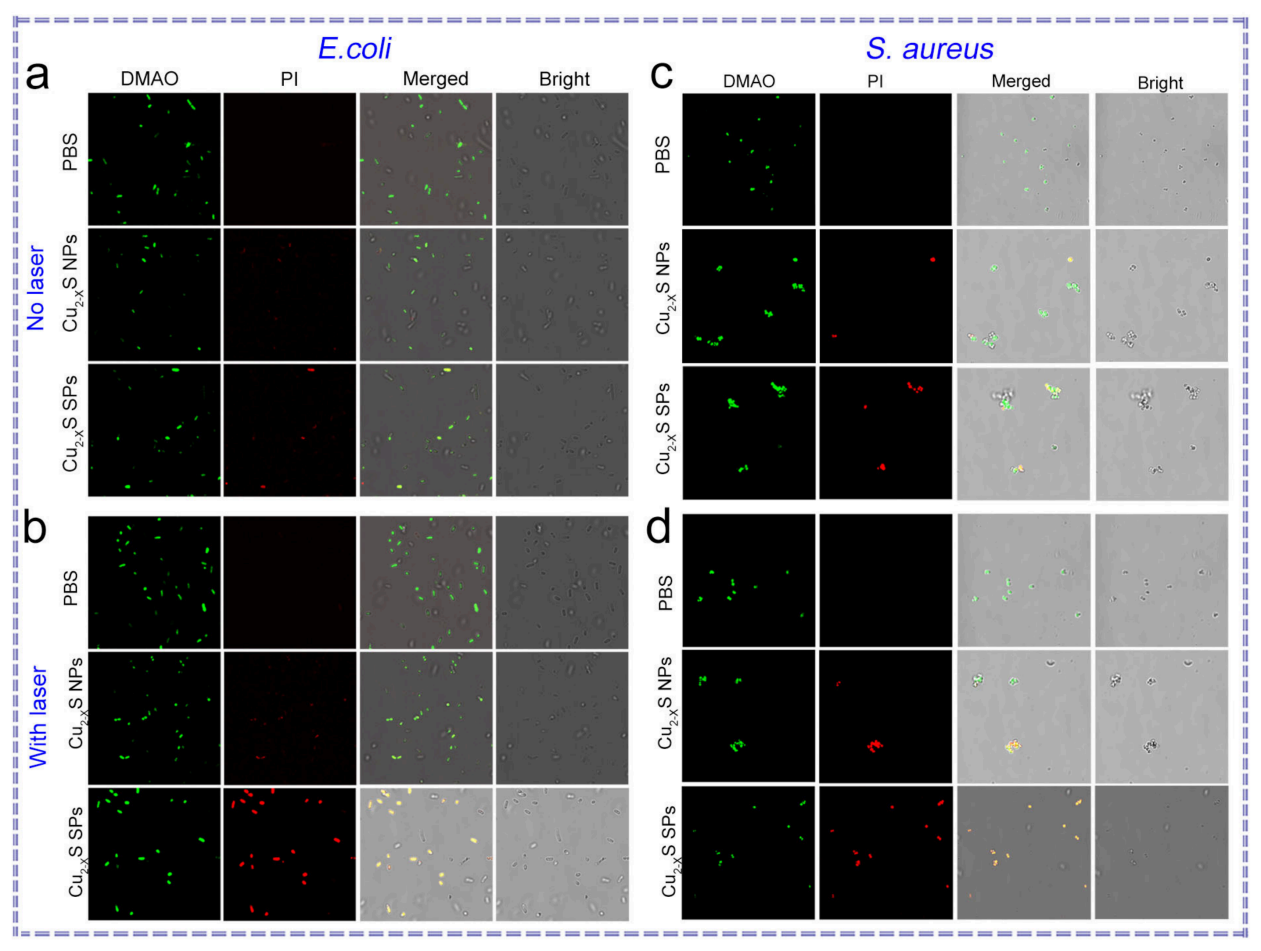
Fluorescence microscopy images of (**a**,**b**) *E. coli* and (**c**,**d**) *S. aureus* after treatment with PBS, Cu_2-x_S NPs, or Cu_2-x_S SPs, with (**a**,**c**) or without 808 nm irradiation (**b**,**d**). Live bacteria were stained with DMAO and showed green fluorescence, while membrane-damaged or dead bacteria were stained with PI and showed red fluorescence; the merged yellow signal indicates bacteria stained by both DMAO and PI.

To further confirm the differences in antibacterial behavior between Cu_2-x_S NPs and Cu_2-x_S SPs, we assessed bacterial membrane integrity of *E. coli* and *S. aureus* using the live/dead bacterial staining kit with DMAO/PI [33]. DMAO (N, N-dimethylaniline N-oxide) is a green fluorescent dye for nucleic acids. It is suitable for both *E. coli* and *S. aureus* and can stain both live and dead cells simultaneously. In contrast, PI (propidium iodide) cannot penetrate the biologically active cytoplasmic membrane and only stain bacteria with compromised membranes. Accordingly, bacteria with intact membranes show strong green fluorescence from DMAO alone, while those with compromised membranes show both green and red signals, appearing yellow in merged fluorescence images. As shown in Figure 5a under dark conditions, *E. coli* treated with the PBS, Cu_2-x_S NPs, and Cu_2-x_S SPs exhibited dominant green fluorescence with minimal yellow signal, indicating that neither nanostructure caused significant membrane damage to *E. coli* in the absence of laser irradiation. After 808 nm laser irradiation (Figure 5d), no obvious yellow fluorescence was observed in the PBS + laser group, confirming that the laser dose alone did not damage bacterial membranes. In contrast, a substantial part of bacteria (approximately 50%) of in the Cu_2-x_S NPs + laser bacterial exhibited yellow fluorescence in the merge group, whereas the Cu_2-x_S SPs + laser group showed an almost complete yellow fluorescence. These results indicate that both Cu_2-x_S SPs and Cu_2-x_S NPs can effectively damage the bacterial membrane of *E. coli* under laser irradiation, with Cu_2-x_S SPs demonstrating a markedly stronger effect.

For *S. aureus*, as shown in Figure 5c, even without laser irradiation, the Cu_2-x_S SPs group exhibited a noticeable increase in yellow fluorescence compared to PBS and Cu_2-x_S NPs group. This finding is consistent with CFU results (Figure 4b) and suggests that the hierarchical Cu_2-x_S SPs induce obvious membrane damage to *S. aureus* even without laser irradiation. Upon 808 nm laser irradiation (Figure 5d), the PBS + laser group showed negligible yellow signal, whereas both nanostructure-treated groups displayed enhanced yellow signals. Notably, the Cu_2-x_S SPs + laser group showed a stronger yellow fluorescence intensity than the Cu_2-x_S NPs + laser group, indicating that Cu_2-x_S SPs have a more significant photothermal destructive effect on the membrane of *S. aureus*.

### 3.4. Antibacterial Mechanism Investigation

**Figure 6:**
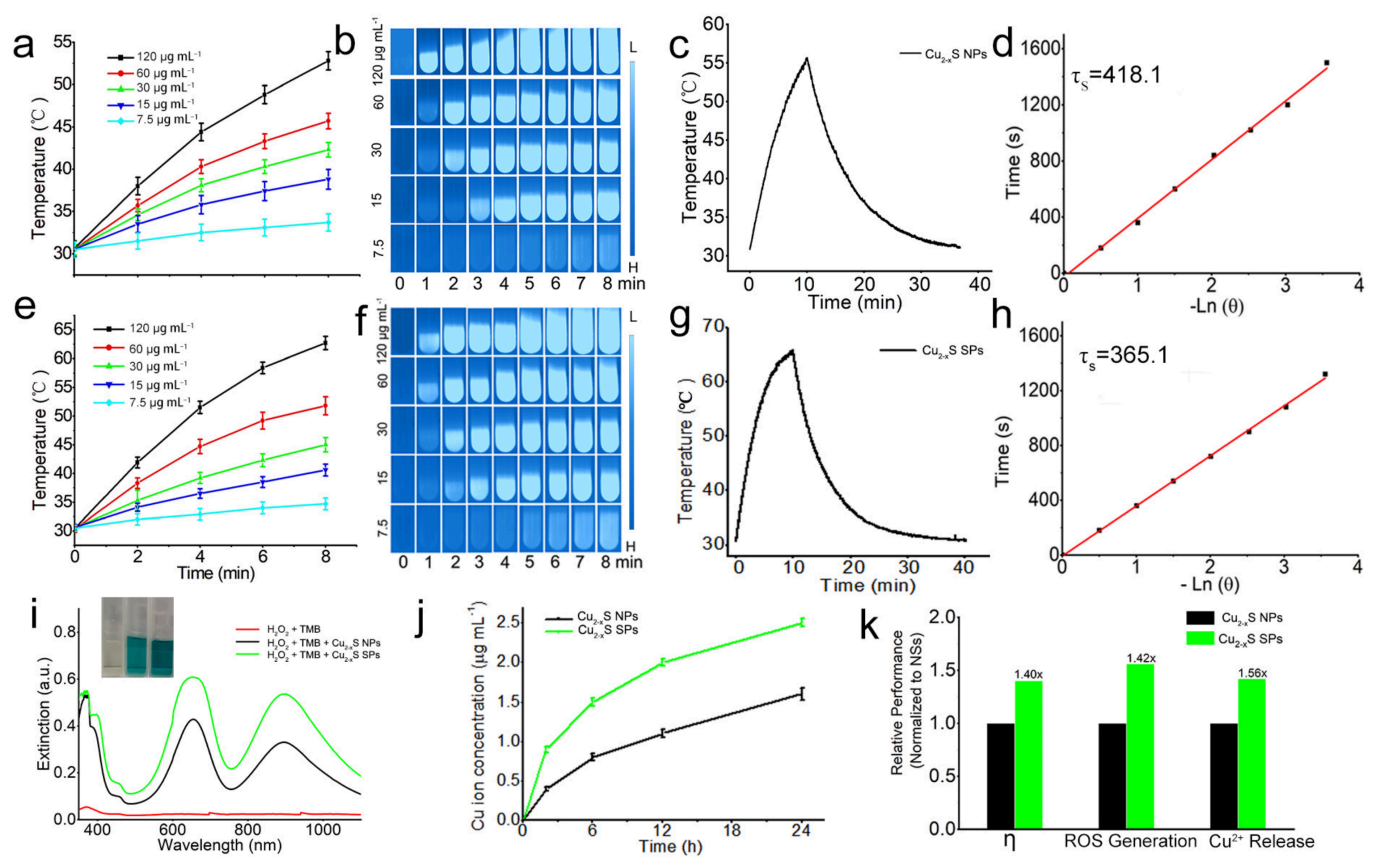
Photothermal and chemical synergistic properties of Cu_2-x_S nanostructures. Photothermal effects of Cu_2-x_S NPs (**a**,**b**) and Cu_2-x_S SPs (**e**,**f**) after 808 nm laser irradiation (0.75 W cm^−2^). Photothermal heating and cooling profiles of Cu_2-x_S NPs (**c**) and Cu_2-x_S SPs (**g**) under the fixed concentration (120 µg mL^−1^). The linear relationships between cooling time and −ln θ of Cu_2-x_S NPs (**d**) and Cu_2-x_S SPs (**h**). (**i**) Extinction spectra of the H_2_O_2_ + TMB + Cu_2-x_S NPs and Cu_2-x_S SPs for 10 min at pH 7.4 (inset: photos of the corresponding solutions). (**j**) The release behavior of Cu^2+^ from Cu_2-x_S NPs and Cu_2-x_S SPs incubated in PBS. (**k**) Comparison between Cu_2-x_S NPs and Cu_2-x_S SPs: photothermal conversion efficiency (η), ROS generation, and Cu^2+^ release.

To clarify the origin of the morphology-dependent antibacterial properties, we systematically investigated the following three synergistic mechanisms: NIR-induced photothermal conversion efficiency, ROS generation, and Cu^2+^ release. The photothermal properties of Cu_2-x_S NPs and Cu_2-x_S SPs were evaluated by monitoring the temperature changes under 808 nm irradiation (0.75 W cm^−2^). As shown in Figure 6a,e, the temperature of both solutions gradually increased with increasing concentration from 7.5 to 120 μg mL^−1^. The data are presented as mean values from three independent measurements. At an identical mass concentration (120 μg mL^−1^), the temperature of the Cu_2-x_S SPs suspension reached a maximum of 62.7 °C after 8 min of irradiation, while that of the Cu_2-x_S NPs system was only 52.8 °C under the same laser conditions. In addition, the stability of Cu_2-x_S NPs and Cu_2-x_S SPs in PBS under the photothermal treatment conditions was evaluated. After 8 min of 808 nm irradiation, both samples retained nearly unchanged UV–vis–NIR absorption spectra and hydrodynamic size distributions (Figure S4), indicating good colloidal stability in PBS without obvious aggregation during laser exposure.

This enhanced photothermal effect is closely related to the morphology. The hierarchical assembly of primary nanoparticles in the Cu_2-x_S SPs can promote multiple internal light scattering, thereby significantly improving light harvesting and non-radiative relaxation. Compared with the relatively flat Cu_2-x_S NPs, the three-dimensional Cu_2-x_S SPs architecture provides more internal interfaces and more complex optical pathways, which can prolong the propagation path of incident NIR light and increase the probability of photon absorption. As a result, more absorbed optical energy is dissipated through non-radiative relaxation, leading to a faster temperature rise and a higher photothermal conversion efficiency. Figure 6b,f show the corresponding infrared thermal images of Cu_2-x_S NPs and Cu_2-x_S SPs, respectively. In these images, whiter colors indicate higher temperatures, while darker colors represent lower temperatures. The results clearly show that Cu_2-x_S SPs exhibit stronger and faster heating properties. Furthermore, the photothermal conversion efficiencies were determined from the cooling profiles following laser shut-off (Figure 6c,d for Cu_2-x_S NPs and Figure 6g,h for Cu_2-x_S SPs). The determined thermal relaxation time (τ_s_) was calculated to be 418.1 s for NPs and 365.1 s for SPs, indicating faster heat dissipation for the latter. The photothermal conversion efficiencies of Cu_2-x_S NPs and Cu_2-x_S SPs are 42.95% and 60.08%, respectively (Figure S4), demonstrating that Cu_2-x_S SPs convert absorbed NIR light into heat more efficiently than Cu_2-x_S NPs. The higher photothermal conversion efficiency of Cu_2-x_S SPs provides a solid foundation for their superior antibacterial performance under NIR irradiation.

Next, the POD-like performances of the Cu_2-x_S NPs and Cu_2-x_S SPs were investigated. The Cu^+^/Cu^2+^ redox couples can catalyze Fenton-like reactions to convert H_2_O_2_ into ·OH. In Figure 6i, after the H_2_O_2_ + TMB + Cu_2-x_S NPs or Cu_2-x_S SPs systems were maintained for 10 min at 37 °C and pH 7.4, both solutions’ color changed from colorless to blue, and the color of the H_2_O_2_ + TMB + Cu_2-x_S SPs system was darker. Both systems showed an obvious characteristic absorbance peaks at 652 nm, corresponding to oxidized TMB. Moreover, the absorbance intensity of the Cu_2-x_S SPs system was higher than that of the Cu_2-x_S NPs. These results indicate that both Cu_2-x_S NPs and Cu_2-x_S SPs can catalyze the H_2_O_2_-mediated generation of ·OH and the subsequent TMB oxidation. These phenomena indicate that Cu_2-x_S SPs exhibit stronger POD-like catalytic activity.

The Cu^2+^ release behaviors of both nanostructures were further compared. Cu_2-x_S NPs and Cu_2-x_S SPs (200 μg each) were dispersed in 2 mL of PBS (pH = 7.4), and dialyzed at 37 °C, respectively. The release medium was collected at different time points (0, 2, 6, 12, and 24 h), and the Cu^2+^ concentrations were determined by ICP-MS. As shown in Figure 6j, Cu_2-x_S SPs exhibited faster Cu^2+^ release kinetics, reaching a cumulative release of 2.5 μg mL^−1^ at 24 h, compared with 1.6 μg mL^−1^ for Cu_2-x_S NPs. This accelerated ion release provides a higher level of bioavailable copper species, which can adsorb onto the bacterial surface, disrupt membrane function, and induce intracellular oxidative stress. Therefore, the faster Cu^2+^ release from the SPs provides an additional contribution to their enhanced antibacterial activity. Together with their superior photothermal performance and stronger ROS generation capability, the increased Cu^2+^ release helps explain why Cu_2-x_S SPs exhibit significantly stronger antibacterial effects than Cu_2-x_S NPs. Overall, as summarized in Figure 6k, the antibacterial ability of Cu_2-x_S SPs is significantly improved relative to that of Cu_2-x_S NPs, which can be mainly attributed to 1.40 times increase in its photothermal conversion efficiency (η), 1.42 times increase in ROS generation, and 1.56 times increase in cumulative Cu^2+^ release.

## 4. Conclusions

In this work, Cu_2-x_S NPs and hierarchical Cu_2-x_S SPs were successfully fabricated via a ligand-directed strategy, enabling a systematic investigation of morphology-dependent antibacterial behavior. By maintaining comparable size, surface ligand environment, and crystal phase between the two nanostructures, the effect of morphology was effectively isolated. The results demonstrate that Cu_2-x_S SPs exhibit significantly enhanced antibacterial activity compared with Cu_2-x_S NPs under both dark and NIR irradiation conditions. This superior performance is attributed to the synergistic effects of enhanced photothermal conversion, increased ROS generation, and accelerated Cu^2+^ release.

Importantly, this study highlights that rational morphology engineering, under well-controlled physicochemical parameters, provides an effective strategy to optimize the antibacterial performance of Cu-based nanomaterials. Compared with conventional approaches that rely on compositional modification or surface functionalization, the present method offers a more controlled platform to elucidate structure–activity relationships. Overall, this work not only provides insights into the role of morphology in antibacterial nanomaterials but also offers valuable guidance for the design of high-performance photothermal antibacterial systems.

## Data Availability

The original contributions presented in this study are included in the article and supplementary material. Further inquiries can be directed to the corresponding authors. Details can be found at: https://susy.mdpi.com/user/assigned/process_form/89d93197ccbd9d75a4981e4a34bbeabc (accessed on 20 April 2026).

## Supplementary Materials

The following supporting information can be downloaded at https://www.mdpi.com/article/10.3390/nano16100636/s1, Table S1: Comparison of representative CuS-based antibacterial systems. Figure S1: TEM of the Cu_2-x_S NPs. Figure S2: The hydrodynamic sizes of the Cu_2-x_S NPs (a) and Cu_2-x_S SPs (b). Figure S3: LSPR bands (a,c) and extinction coefficient at 808 nm (b,d) of the Cu_2-x_S NPs and Cu_2-x_S SPs, respectively. Figure S4: Stability of Cu_2-x_S NPs and SPs in PBS before and after 8 min of 808 nm laser irradiation. Absorption spectra of Cu_2-x_S NPs (a) and SPs (c), respectively. DLS size distributions of Cu_2-x_S NPs (b) and SPs (d), respectively. Calculation of the photothermal conversion efficiency.
