# Supplementary material for: Morphology-Dependent Antibacterial Activity of Cu2-xS Nanostructures: Nanoplates Versus Superparticles
Source: Nanomaterials (Basel). 2026 May 20;16(10):636. doi: 10.3390/nano16100636 (PMC13209337; doi:10.3390/nano16100636)
Supplement: Supplementary file 1 [file nanomaterials-16-00636-s001.zip › nanomaterials-4302255-supplementary.pdf]

Supporting Information

Morphology-Dependent Antibacterial Activity of $Cu_{2-x}S$ Nanostructures: Nanoplates *versus* Superparticles

Hui Zhu[1], Mengzhe Zhao[2], Yang Chao[2], Jun Yao[1]*, Qin Yu[1], *and Na Sun[3]**

1. School of Modern Fashion, Anhui University of Applied Technology, Hefei City 230011, China

2. Key Laboratory of Functional Molecular Solids, Ministry of Education College of Chemistry and Materials Science, Anhui Normal University, Wuhu 241000, China.

3. Huzhou Key Laboratory of Green Energy Materials and Battery Cascade Utilization, School of Intelligent Manufacturing, Huzhou College, Huzhou, 313000, China.

Table S1.Comparison of representative CuS-based antibacterial systems, mainly focused on morphology-dependent performance, together with the present work

CuS-based system	Main feature	Size (TEM)	Antibacterial mechanism	Advantage	Limitation	Ref
Ultrasmall CuS nanodots (NDs) vs larger CuS nanoparticles (NPs)	Particle size effect	nanodots (6 nm), nanoparticles (20 nm)	Cu^{2+} release + ROS generation + photothermal effect	Very small size, large specific surface area, enhanced bacteria–particle interaction, strong bactericidal activity	The surface ligand was not controlled, so the interference of ligand effects on antibacterial activity cannot be excluded.	8
CuS microspheres (MSs), nanosheets (NSs), and nanoparticles (NPs)	Morphology-dependent antibacterial activity	microspheres (2.87 μm), nanosheets (56.10 nm), and nanoparticles (22.43 nm)	Light-induced ROS + NIR photothermal effect	Direct comparison of microscale vs nanoscale CuS; confirms that nanosized/sheet-like structures are more effective	Three morphologies show large size variation and poor monodispersity.	18
Cysteine-coated amorphous porous CuS-C nanoparticles	Porous/amorphous structure effect	250 nm	Enhanced photothermal conversion + ROS-assisted killing under light irradiation	Porous morphology improves light absorption; amorphous structure promotes ROS generation; cysteine coating improves biocompatibility	Antibacterial effect depends on external irradiation; no obvious killing under light-free conditions	21
Shape-controlled CuS nanocrystals (sphere, cube, cuboctahedron ,T-octahedron)	Shape-dependent photothermal/ photodynamic behavior	Sphere (180 nm), cube (254 nm), cubctahedron (246 nm) and T-octahedron (240 nm)	Photothermal + photodynamic effects	Provides mechanistic evidence that crystal shape strongly affects photoactivity; useful for rational morphology design	Focused mainly on photo-responsive performance; direct antibacterial comparison was limited	16
Positively charged vancomycin-functionalized CuS nanoparticles (cCuS@Van)	Representative surface-functionalized CuS system	30 nm-50 nm,	Bacterial targeting + antibiotic-assisted binding + NIR-II photothermal killing	Strong bacterial targeting, low effective dose, deep tissue penetration under 1064 nm irradiation	More complex design than bare CuS; antibacterial spectrum may depend on surface functionalization	14
$Cu_{2-x}S$ nanoplates (NPs) and hierarchical superparticles (SPs)	Morphology-regulated antibacterial comparison under matched physicochemical parameters	nanoplates (16.03 nm) and superparticles (16.48 nm)	Enhanced photothermal conversion + increased ROS generation + accelerated Cu^{2+} release	Enables a clearer evaluation of the morphology effect by keeping size, surface ligands, and crystal phase comparable; no complicated surface	Further studies on long-term biosafety and practical application conditions are still needed	This work

functionalization or compositional modification required; provides direct structure–activity insight for Cu-based antibacterial nanomaterials

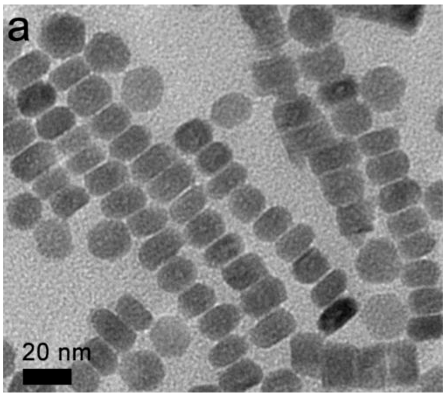

Figure S1. TEM of the $Cu_{2-x}S$ NPs.

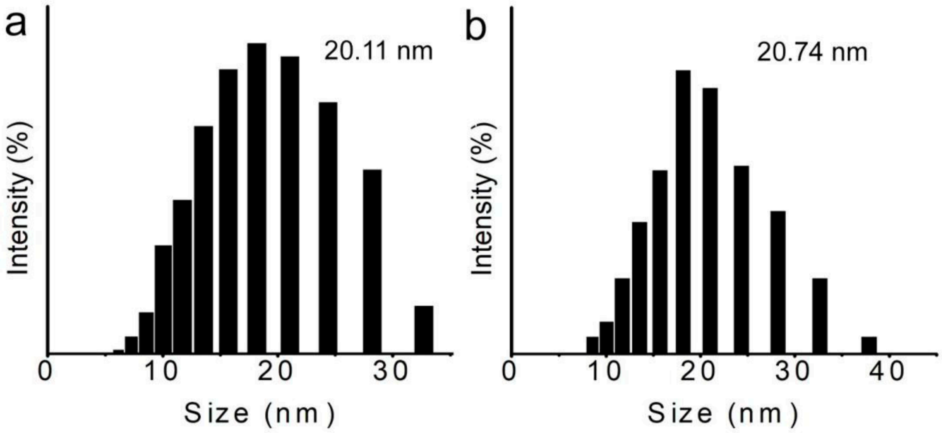

Figure S2.The hydrodynamic sizes of the $Cu_{2-x}S$ NPs (a) and $Cu_{2-x}S$ SPs (b).

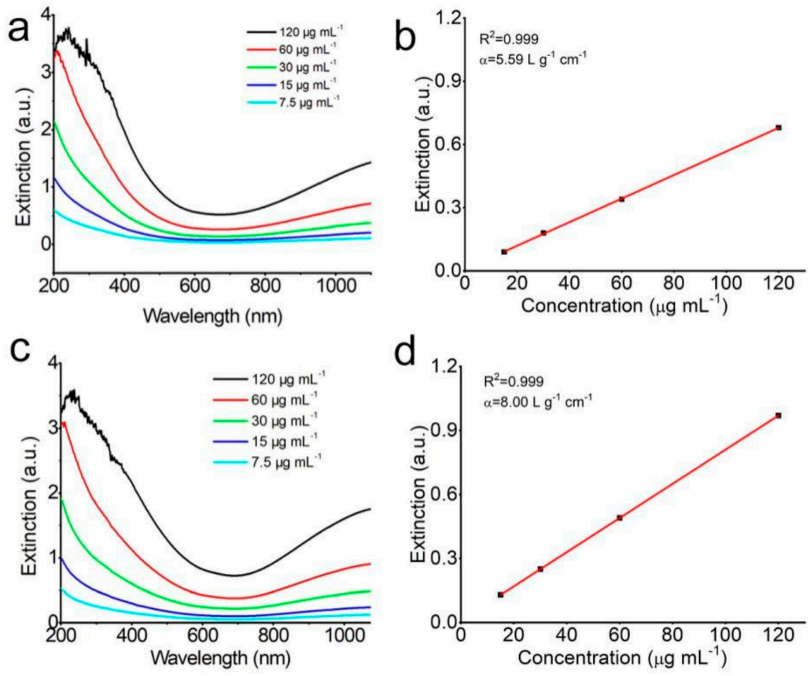

Figure S3. LSPR bands (a, c) and extinction coefficient at 808nm (b, d) of the $Cu_{2-x}S$ NPs and $Cu_{2-x}S$ SPs, respectively.

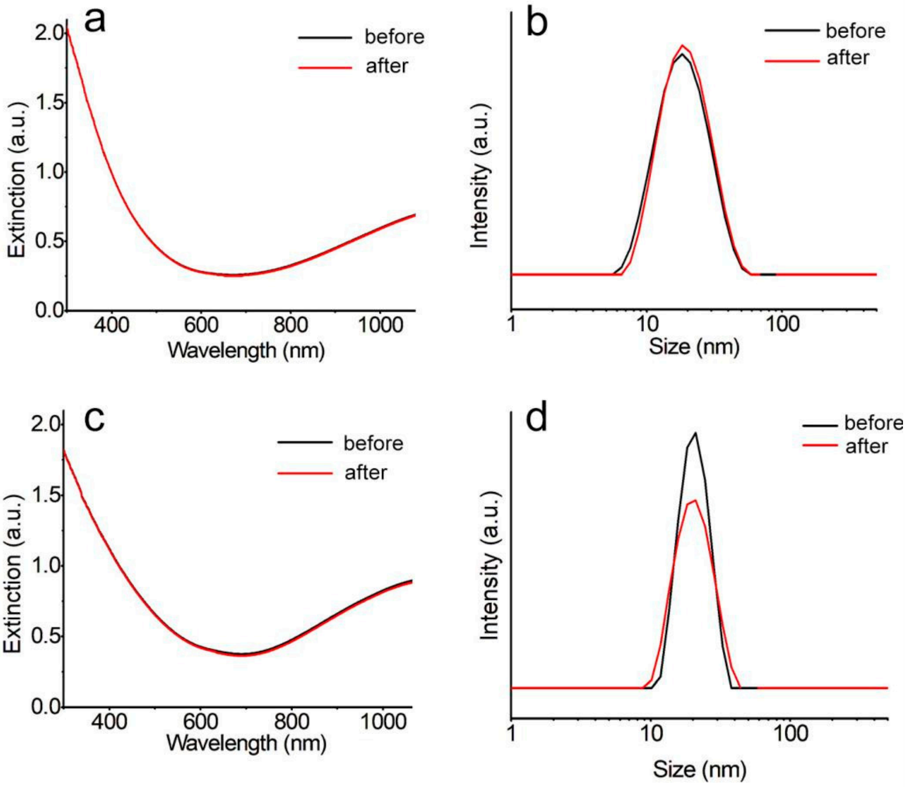

Figure S4. Stability of $Cu_{2-x}S$ NPs and SPs in PBS before and after 8 min of 808 nm laser irradiation. UV–vis–NIR absorption spectra of $Cu_{2-x}S$ NPs (a) and SPs (c), respectively. DLS size distributions of $Cu_{2-x}S$ NPs (b) and SPs (d), respectively.

2. **Calculation of the photothermal conversion efficiency**

The photothermal conversion efficiencies (η) were calculated using following equations.

$$\eta = \frac{h\, s\, (T_{max}\text{-}T_{max,H_2O})}{I\,(1\text{-}10^{-A_{808}})} \qquad (S1)$$

$$\tau_s = \frac{m_D\, C_D}{h\, S} \qquad (S2)$$

$$\theta = \frac{T\text{-}T_{surr}}{T_{max}\text{-}T_{surr}} \qquad (S3)$$

$$t = -\tau_s \ln \theta \qquad (S4)$$

$$\eta = \frac{m\, c\, (T_{max}\text{-}T_{max,H_2O})}{I\,(1\text{-}10^{-A_{808}})\, \tau_s}$$

$$\eta_{NSs} = \frac{1\times4.2\times(55.46\text{-}30.09)}{0.75\times(1\text{-}10^{-0.68})\times418.1}\times100\%$$
$$=42.95\%$$

$$\eta_{SPs} = \frac{1\times4.2\times(65.06\text{-}30.09)}{0.75\times(1\text{-}10^{-0.97})\times365.1}\times100\%$$
$$=60.08\%$$

Where m is the mass of the aqueous dispersion (1.0 g), C is the specific heat capacity of the solvent (water, 4.2 J g^{-1} K^{-1}), and t is the cooling time after the laser is turned off, τ_s represents the system time constant for heat transfer, θ is the dimensionless driving force temperature, and T is the temperature of the solution at cooling time t. The time constant τ_s can be determined by the linear fit of the cooling time (t) versus the negative natural logarithm of the dimensionless temperature (−ln θ).
